# Supplementary material for: Identification and functional analysis of non-coding regulatory small RNA FenSr3 in Bacillus amyloliquefaciens LPB-18
Source: PeerJ. 2023 May 15;11:e15236. doi: 10.7717/peerj.15236 (PMC10194069; doi:10.7717/peerj.15236)
Supplement: Supplemental Information 4 [file peerj-11-15236-s004.zip › KO/CK-vs-T1_map/map04727.html]

KEGG PATHWAY: GABAergic synapse - Reference pathway


|  |  |
| --- | --- |
| **GABAergic synapse - Reference pathway** |  |

[
Pathway menu
| Organism menu
| Pathway entry
| Show description
| User data mapping
]

Gamma aminobutyric acid (GABA) is the most abundant inhibitory neurotransmitter in the mammalian central nervous system (CNS). When released in the synaptic cleft, GABA binds to three major classes of receptors: GABAA, GABAB, and GABAC receptors. GABAA and GABAC receptors are ionotropic and mediate fast GABA responses by triggering chloride channel openings, while GABAB receptors are metabotropic and mediate slower GABA responses by activating G-proteins and influencing second messenger systems. GABAA receptors, the major sites for fast inhibitory neurotransmission in the CNS, are regulated by phosphorylation mechanisms, affecting both their functional properties and their cell surface mobility and trafficking. GABA release by the presynaptic terminal is negatively regulated by GABAB autoreceptors, and is cleared from the extracellular space by GABA transporters (GATs) located either on the presynaptic terminal or neighboring glial cells.

|  |  |  |
| --- | --- | --- |
| Reference pathway | 184% 150% 122% 100% 82% 67% 55% | 图片下载 |
